# Access to quality-assured artemisinin-based combination therapy and associated factors among clients of selected private drug outlets in Uganda

**DOI:** 10.1186/s12936-024-04956-5

**Published:** 2024-04-30

**Authors:** Moses Ocan, Loyce Nakalembe, Caroline Otike, Tayebwa Mordecai, Joan Birungi, Sam Nsobya

**Affiliations:** 1 Department of Pharmacology & Therapeutics, Makerere University College of Health Sciences, P. O. Box 7072, Kampala, Uganda; 2 Department of Pharmacology, Soroti University, P. O. Box 211, Soroti, Uganda; 3 Data Department, Joint Clinical Research Centre, Lubowa, P. O Box 10005, Kampala, Uganda; 4 Makerere University College of Health Sciences Grants Office, P. Box 7072, Kampala, Uganda; 5 Department of Molecular Biology and Immunology, Makerere University College of Health Sciences, P. O. Box 7072, Kampala, Uganda; 6 Department of Pathology, College of Health Sciences, Makerere University, P.O. Box 7072, Kampala, Uganda

**Keywords:** Quality assured, Artemisinin agents, Malaria, Pharmacies, Uganda

## Abstract

**Background:**

Malaria treatment in sub-Saharan Africa is faced with challenges including unreliable supply of efficacious agents, substandard medicines coupled with high price of artemisinin-based combinations. This affects access to effective treatment increasing risk of malaria parasite resistance development and adverse drug events. This study investigated access to quality-assured artemisinin-based combination therapy (QAACT) medicines among clients of selected private drug-outlets in Uganda.

**Methods:**

This was a cross sectional study where exit interviews were conducted among clients of private drug outlets in low and high malaria transmission settings in Uganda. This study adapted the World Health Organization/Health Action International (WHO/HAI) standardized criteria. Data was collected using a validated questionnaire. Data entry screen with checks was created in Epi-data *ver* 4.2 software and data entered in duplicate. Data was transferred to STATA *ver* 14.0 and cleaned prior to analysis. The analysis was done at 95% level of significance.

**Results:**

A total of 1114 exit interviews were conducted among systematically sampled drug outlet clients. Over half, 54.9% (611/1114) of the participants were males. Majority, 97.2% (1083/1114) purchased an artemisinin-based combination anti-malarial. Most, 55.5% (618/1114) of the participants had a laboratory diagnosis of malaria. Majority, 77.9% (868/1114) of the participants obtained anti-malarial agents without a prescription. Less than a third, 27.7% (309/1114) of the participants obtained a QAACT. Of the participants who obtained QAACT, more than half 56.9% (173/309) reported finding the medicine expensive. The predictors of accessing a QAACT anti-malarial among drug outlet clients include type of drug outlet visited (aPR = 0.74; 95%CI 0.6, 0.91), not obtaining full dose (3-day treatment) of ACT (aPR = 0.49; 95%CI 0.33, 0.73), not finding the ACT expensive (aPR = 1.24; 95%CI 1.03, 1.49), post-primary education (aPR = 1.29; 95%CI 1.07,1.56), business occupation (aPR = 1.24; 95%CI 1.02,1.50) and not having a prescription (aPR = 0.76; 95%CI 0.63, 0.92).

**Conclusion:**

Less than a third of the private drug outlet clients obtained a QAACT for management of malaria symptoms. Individuals who did not find artemisinin-based combinations to be expensive were more likely to obtain a QAACT anti-malarial. The Ministry of Health needs to conduct regular surveillance to monitor accessibility of QAACT anti-malarial agents under the current private sector copayment mechanism.

## Background

The artemisinin-based combination anti-malarial agents are the current cornerstone of malaria treatment globally [1]. The WHO recommends artemisinin-based combination therapy (ACT) for treatment of uncomplicated malaria and artesunate for severe malaria [2]. In Uganda, first-line and alternative first-line for uncomplicated malaria is artemether–lumefantrine (AL) and artesunate–amodiaquine (AQ), respectively. Artesunate is the preferred first-line agent for severe malaria [2, 3]. The Ministry of Health recommends dihydroartemisinin–piperaquine (DHP) as second-line agent for uncomplicated malaria and artemether as second-line agent for severe malaria [2]. ACT remains an efficacious anti-malarial treatment across sub-Saharan Africa [1]. However, access to these agents is affected by unreliable supply (availability), and cost (affordability) especially in sub-Sahara African countries [4, 5].

The private sector provides the first point of care for over 50% of the population in Uganda [6]. The sector is key in the fight against malaria with most patients accessing their antimalarial agents in private drug outlets [7]. A previous study by Ocan et al*.* [8] reported that over 60% of the population in northern Uganda access antimalarial agents without a prescription. Unreliable supply of medicines common in public health facilities and cost in the private sector are the leading barriers of access to anti-malarial agents [9]. However, the private sector continues to play a pivotal role in the provision of healthcare services in most low-and middle-income countries (LMICs). Almost 3.8 billion treatment courses of artemisinin-based combinations were delivered globally by manufacturers in 2010–2012, with about 1.2 billion courses going to the private sector [1]. In 2021 alone some 242 million courses of artemisinin-based combinations were distributed by national malaria programmes, of which 97% were in sub-Saharan Africa [1].

A previous study done in Uganda reported cases where malaria patients in the private sector are not able to purchase a full dose of ACT anti-malarial agents [10]. This potentially predisposes patients to inappropriate treatment practices such as taking suboptimal dose of antimalarial agents. Therefore, over the counter access of anti-malarial agents in private drug outlets is likely to further exacerbate the risk of inadequate treatment as most patients fail to afford a full dose of artemisinin-based combination.

The affordable medicines facility for malaria (AMF-m) programme coordinated by the global fund was piloted in 2011 in nine sub-Saharan African countries, including Uganda [11, 12]. The programme intended to improve availability, affordability and crowd out artemisinin monotherapies in the drug market [4]. A subsequent programme, the Copayment mechanism was introduced after the AMF-m pilot period under the same objectives in 2013 [4]. Under the copayment mechanism, Uganda is currently providing 70% subsidy on QAACTs supplied in the private sector [10]. The QAACT are artemisinin-based combinations supplied under the AMF-malaria pilot programme and subsequently the copayment mechanism having subsidized prices with the packaging material carrying a green leaf logo [4]. However, there is no readily available information to show whether the increased availability and use of QAACT during the AFM-m pilot programme have been sustained under the copayment mechanism. This study conducted exit interviews among clients of selected private drug outlets to assess the extent of access to QAACTs for management of symptoms of malaria across the country.

## Methods

### Study design and setting

This was a cross-sectional study conducted in selected private drug outlets in low (Kabale and Mbarara districts) and high (Apac and Tororo districts) malaria transmission settings between June-December 2021. Access in this study refers to purchasing/obtaining an anti-malarial agent by clients of private drug outlets for management of symptoms of malaria.

### Sample size, private drug outlet selection and sampling

In each study district (Tororo, Apac, Mbarara and Kabale), a list of private drug outlets was compiled using the private drug out let registry of the National drug authority. In addition, a census was taken to identify all the private drug outlets (pharmacies and drug shops) in each district prior to data collection. The drug outlets identified through the National drug authority register and the census were then contacted for inclusion into the study and data collected from those that consented to participate in the study. Private drug outlets in this study are defined as for-profit licensed establishments that sale and/or dispense medicines. Following World Health Organization/Health Action International (WHO/HAI) standardized criteria [13], a minimum of 30 drug outlet clients were interviewed from each of the private drug outlets visited in the four districts. Two research assistants, a pharmacist (KJ) and nurse (RK) conducted the exit interviews. Each of the research assistants separately visited different drug outlets in the study districts. At each drug outlet, prior to data collection the research assistants introduced themselves to the pharmacist/dispenser. The research assistants then waited for potential study participants outside the drug outlet. This was repeated in each of the study districts, where the research assistants moved from one drug outlet to the next until when the required sample size was achieved. Using systematic random sampling every 3rd private drug outlet client was contacted for enrolment into the study.

### Data collection

Exit interviews conducted using a questionnaire were used to collect data from drug outlet clients who reported to have obtained an anti-malarial agent. The questionnaire was adapted from the World Health Organization/Health Action International (WHO/HAI) standardized tool. Additionally, information from previous studies [14, 15] was also used in developing the tool. The draft tool was pre-tested among drug outlet clients in Kampala city and the information obtained was used to further adjust the questionnaire. The tool collected data on the following areas, (i) socio-demographic characteristics (age, sex, occupation and level of education), (ii) reason for visiting the drug outlet, (iii) possession of a prescription, (iii) type of antimalarial medicine obtained, (iv) QAACT purchased, (v) cost of artemisinin-based combination therapy (ACT), (vi) dose of artemisinin-based combination obtained, (vii) having malaria diagnosis, (viii) location of drug outlet (malaria transmission setting), (ix) drug outlet type, (x) source of information on anti-malarial purchased, and xi) awareness on QAACT. The presence of AMFm logo (‘Green leaf’) on the primary package was used to identify a QAACT. The research assistants were trained on the data collection tool and study protocol prior to field data collection.

During each data collection day, the lead researcher reviewed all the filled data collection forms for quality control checks. Any discrepancies in the filled data collection tools were rectified through discussion with the data collectors and contacting the respondents.

### Data management and analysis

Data was entered in EpiData *version* 4.2.0 and exported to STATA *version* 14.0 for cleaning and analysis. The artemisinin-based combinations obtained by the drug outlet clients were classified as QAACT and non-QAACT. The list of artemisinin-based combinations that the patients obtained from the drug outlets were matched against the list of QAACT approved by the Ministry of Health for distribution in Uganda. Anti-malarial agents were summarized using frequencies and proportions. Access to QAACT anti-malarial agents across malaria transmission settings was compared using Fisher’s exact test. Price of anti-malarial agents was summarized using median and interquartile range. The factors associated with access to QAACTs in private drug outlets were assessed using modified Poisson regression at bivariate level to establish the crude relationship between the predictors and the outcome variable, and multivariate levels to assess the adjusted relationship between the predictors and the outcome variables. The factors that had P-value of 0.2 and below from the bivariate analysis were included in the multivariable regression analysis. Statistical significance was determined at 5% level of significance.

## Results

### Socio-demographic characteristics of study participants

Exit interviews were conducted among a total of 1114 participants from 37 drug outlets visited in the four districts. Of these 54.9% (611/1114) were males, and over a third 37.7% (420/1114) were peasant farmers. The average age was 31.5 ± 0.5 years. The median cost of ‘Green leaf ACT’ (QAACT) in the drug outlets was USD 0.97 (0.83, 1.1). The majority, 82.9% (924/1114) of study participants who visited the drug outlets had fever, headache, fatigue, and joint pains (Table 1).

**Table 1:** Description of the study participants (N = 1114) visiting drug outlets June–Dec 2021

Characteristic	Description	Frequency, n (%)	High malaria transmission setting n (%)	Low malaria transmission setting n (%)	*p-*value
Gender	Male	611 (54.9)	565 (50.7)	46 (4.2)	0.908
	Female	503 (45.1)	467 (41.9)	36 (3.2)	
Drug outlet	Pharmacy	580 (52.1)	531 (47.7)	49 (4.4)	0.168
	Drug shop	534 (47.9)	501 (45)	33 (2.9)	
Reason for visiting drug outlet today	Had fever, headache, fatigue, and joint pains	924 (82.9%)	848 (76.1)	76 (6.8)	0.014
	To consult for what medicines to buy	123 (11)	110 (9.9)	13 (1.1)	0.145
	To buy anti-malarial medicine	587 (52.7)	544 (48.8)	43 (3.9)	1.000
	Others	745 (66.8)	690 (61.9)	55 (4.9)	1.000
Type of anti-malarial agent purchased	ACT	1083 (97.2)	1008 (90.5)	75 (6.7)	0.006
	Non-ACT	31 (2.8)	24 (2.2)	7 (0.6)	
Having malaria lab. diagnosis	Yes	612 (54.9)	574 (51.5)	38 (3.4)	0.108
	No	502 (45)	458 (41.1)	44 (3.9)	
Type of malaria diagnostic test	RDT	430 (38.6)	419 (37.6)	11 (1.0)	< 0.001
	Microscopy	179 (16.0)	153 (13.7)	26 (2.3)	
	Both RDT and microscopy	3 (0.3)	2 (0.2)	1 (0.1)	
	Missing	10 (0.9)	6 (0.5)	4 (0.4)	
Have a prescription	Yes	246 (22.1)	232 (20.8)	14 (1.3)	0.332
	No	868 (77.9)	800 (71.8)	68 (6.1)	
Occupation	Peasant farmers	420 (37.7)	392 (35.2)	28 (2.5)	0.039
	Business	255 (22.9)	238 (21.4)	17 (1.5)	
	Formal employment	144 (12.9)	125 (11.2)	19 (1.7)	
	Others	295 (26.5)	277 (24.9)	18 (1.6)	
Level of education	No formal education	260 (23.3)	251 (22.5)	9 (0.8)	0.002
	Primary education	346 (31.1)	326 (29.3)	20 (1.8)	
	Post primary	299 (26.8)	270 (24.2)	29 (2.6)	
	Tertiary education	209 (18.8)	185 (16.6)	24 (2.2)	
Unit price of ACT (USD)	Median (25th, 75th percentile)	1.1 (0.83, 1.1)	1.1 (0.83, 1.4)	1.4 (1.25, 3.33)	< 0.001

*RDT* Malaria rapid diagnostic test, *ACT* Artemisinin-based combination therapy, *IQR* interquartile range, *n* sample size

### Access to quality assured artemisinin combination therapy (QAACT) antimalarial agents

Majority, 97.2% (1083/1114) of the participants purchased ACT anti-malarial agents. Less than a third of the drug outlet clients (27.7%:309/1114) purchased QAACTs. Over half, 62.5% (193/309) of the QAACTs were accessed from private pharmacies. A majority, 72.8% (225/309) of the study participants who purchased QAACTs did not have a prescription. More than a half, 56.9% (173/309) of the participants reported finding QAACT agents expensive. A majority, 82.5% (255/309) of the respondents reported being able to purchase a full dose of QAACTs. Among participants who obtained QAACT, Combiart brand was the most, 45.1% (139/308) perceived as highly efficacious. Over half, 57.5% (177/309) of the participants who obtained QAACT had a laboratory diagnosis of malaria. Most, 82.7% (253/309) of the respondents obtained full oral dose of QAACTs (3-day treatment). Of the participants who did not receive a full 3-day dose of the artemisinin-based combinations, on average purchased seven tablets (less than 1 day’s dose) instead of the recommended 24 tablets for the full 3-day adult dose.

The most common brands of artemisinin-based combinations obtained by the study participants include Lariact 15.6% (156/1114), Artefan 14% (152/1114), and Co-mether 12.8% (139/1114). The choice of which ACT to purchase from the drug outlets was guided by the perceived efficacy, 75.8% (819/1114) and cost/affordability, 18.1% (196/1114).

For participants who did not have a prescription, purchasing a particular ACT was mostly based on previous experience 48.2% (418/1114), and recommendation from the pharmacist/dispenser 42% (368/1114).

### Self-reported efficacy of artemisinin-based combination therapy (ACT) by the private drug outlet clients

Nearly a third, 26.6% (296/1114) of the study participants reported persistence of malaria symptoms even after taking a full oral dose (3-day treatment) of ACT. The reported actions taken when malaria symptoms fail to resolve following ACT include going back to the health facility for further management 40.6% (119/296), purchasing a different artemisinin-based combination 37.7% (110/296) and purchasing the same artemisinin-based combination 22.5% (65/296). On average study participants reported purchasing artemisinin-based combinations for managing malaria symptoms at least twice (2) in the last three months prior to survey date.

### Determinants of access to QAACTs in private drug outlets in Uganda

From bivariate analysis, the factors significantly associated with accessing QAACTs among the study participants include drug shop (*p* < 0.001), attaining post primary level of education (p = 0.003), being a businessperson (p < 0.001), not purchased full oral dose of ACTs (3-day treatment) (*p* = 0.004), not finding the ACT expensive (*p* < 0.001) and not having a prescription (*p* = 0.007).

From multivariable analysis, individuals who obtained their ACT anti-malarials from drug shops had 26% decreased prevalence of purchasing a QAACT compared to those who went to the pharmacies (aPR = 0.74; 95%CI 0.60–0.91). Participants who did not purchase full oral dose of ACT were 51% less likely to purchase a QAACT compared to those who purchased full oral dose of ACT (aPR = 0.49; 95%CI 0.33,0.73). Drug outlet clients who did not find the ACT expensive were 24% more likely to purchase a QAACT compared to those who found ACT expensive (aPR = 1.24; 95%CI 1.03, 1.49). Individuals who did not have a prescription were 24% less likely to purchase QAACTs from the drug outlets compared to those who had a prescription (aPR = 0.76; 95%CI 0.63, 0.92). Participants who attained post primary education were 29% more likely to purchase a QAACT compared to those with no formal education (aPR = 1.29; 95%CI 1.07, 1.56). Business respondents were 24% more likely to purchase QAACT compared to peasant farmers (aPR = 1.24; 95%CI 1.02, 1.50) (Table 2).

**Table 2:** Determinants of access to QAACTs in private drug outlets in Uganda

Characteristic	Description	Proportion n (%)	cPR (95%CI)	aPR (95%CI)	*p-*value
Gender	Male	594 (55.8)	1	1	
	Female	136 (44.2)	0.96 (0.99, 1.01)	0.96 (0.80, 1.15)	0.674
Education	No formal education	63 (20.5)	1	1	
	Primary education	82 (26.6)	0.98 (0.74, 1.30)	0.92 (0.70–1.22)	0.577
	Post primary	108 (35.1)	1.49 (1.14, 1.93)	1.29 (1.07, 1.56)	0.008
	Tertiary education	55 (17.9)	1.12 (0.82, 1.53)	1.10 (0.75, 1.61)	0.621
Occupation	Peasant farmers	93 (30.2)	1	1	
	Business	91 (29.6)	1.61 (1.26, 2.05)	1.24 (1.02, 1.50)	0.033
	Formal employment	37 (12.0)	1.20 (0.87, 1.67)	1.12 (0.75, 1.67)	0.566
	Others	87 (28.3)	1.34 (1.05, 1.73)	1.20 (0.92, 1.56)	0.177
Drug outlet	Pharmacy	192 (62.3)	1.0	1.0	
	Drug shop	116 (37.7)	0.67 (0.55, 0.81)	0.74 (0.60, 0.91)	0.004
ACT for 3-days	Yes	252 (82.6)	1.0	1.0	
	No	53 (17.4)	0.68 (0.53, 0.89)	0.49 (0.33, 0.73)	< 0.001
ACTs expensive	Yes	172 (56.8)	1.0	1.0	
	No	131 (43.2)	1.47 (1.22, 1.7)	1.24 (1.03, 1.49)	0.022
Ever heard of QAACTs	Yes	64 (20.8)	1.0	1.0	
	No	244 (79.2)	1.12 (0.88, 1.41)	1.14 (0.91, 1.44)	0.260
Had a prescription	Yes	83 (27.0)	1.0	1.0	
	No	225 (73.0)	0.76 (0.62, 0.94)	0.76 (0.63, 0.92)	0.005
Unit price for ACTs,	Median (25th, 75th)	0.97 (0.83, 1.11)	1.0	1.0	–

## Discussion

This study found less than a third of the participants purchased QAACT anti-malarial medicines from private drug outlets. This finding is like that of a previous study by the ACT Watch Group [4], which reported that QAACT accounted for less than 50% of anti-malarial agents in private for-profit drug outlets in all AMF-m pilot countries including Uganda. This could explain the low access to QAACT anti-malarial agents among private drug outlet clients found in the current study. This is especially important as the private sector is the first point of care in most communities in sub-Saharan Africa [16]. The low access to QAACTs among malaria patients found in this study may indicate high use of non-QAACTs antimalarial agents in the communities. With the reported prevalence of substandard artemisinin-based combinations in most LMICs including Uganda, the use of non-QAACTs anti-malarial agents potentially exposes malaria patients to the risk of inadequate treatment [17]. Further worsening the current challenge of artemisinin resistance emergence and spread in the country [18, 19].

The low access to subsidized QAACT found in this study is an indicator of the challenges in implementation of government programmes especially in low-and middle-income countries. A previous study by Tougher et al*.* [5] reported lack of full-scale public awareness campaigns (supporting interventions) as a main barrier in implementation of AMFm and copayment mechanism. However, over 90% of the respondents obtained non-QAACTs from the drug outlets for management of malaria symptoms. This is like findings of a previous study by Kibira et al. [10] that reported 100% availability of ACTs in the private sector. Despite the wide availability of ACTs in private sector, cost remains a major barrier to their access [10]. The findings of the current study highlight the need for government to strengthen implementation of interventions such as private sector copayment mechanism.

Most of the participants who obtained QAACTs from private drug outlets did not have a prescription. This is like findings of a previous study that reported high prevalence of use of over-the-counter ACT in communities of northern Uganda [8]. Accessing ACT over-the-counter exposes patients to the risk of suboptimal and most commonly inappropriate treatment practices like obtaining less than recommended dose of anti-malarial agent. In the current study, majority of the participants obtained on average seven tablets of artemisinin-based combination antimalarial which is less than one day’s adult dose. Such practices could potentially exacerbate artemisinin resistance development and spread [17].

Close to a third of the participants reported no improvement despite taking a full oral dose of ACT (3-day treatment). The lack of improvement could be an indicator of potential parasite resistance among the malaria patients. This is supported by findings of previous studies in Uganda that reported prevalence of *Plasmodium falciparum* parasite resistance against artemisinin agents [19, 20]. The development and spread of resistance to ACT threatens to increase the risk of malaria related mortality across malaria affected regions due especially to lack of affective therapeutic alternative. Previously the widespread resistance to chloroquine resulted in more than doubling of malaria mortality especially in sub-Saharan Africa [21]. There is thus need for establishment of interventions to mitigate widespread parasite resistance against artemisinin agents in the country.

In this study participants who reported finding ACT not to be expensive were 28% more likely to obtain a QAACT. This is like findings of previous studies [4, 5] that reported cost as a barrier to access to anti-malarial agents. Programs which target making anti-malarial agents affordable especially in the private sector are key in the fight against malaria. In Uganda, the copayment mechanism is being implemented with the aim of making QAACTs more affordable to communities as one of its core pillars [4]. However, over half of the participants reported that they found QAACTs in the drug outlets expensive and thus unable to purchase a full course of treatment. This potentially leads to use of suboptimal dose of the QAACTs for malaria treatment [17] which could predispose to parasite resistance development [17]

In this study, the participants who were less likely to obtain QAACT from private drug outlets included those who; purchased less than 3-day dose of ACT, lacked a prescription and drug shop clients. While respondents who did not find ACT to be expensive were more likely to purchase a QAACT anti-malarial agent. These findings are like those of a previous study that reported cost as a barrier to access to anti-malarial agents in the private sector [10]. The use of non-QAACT coupled with inappropriate practices such as use of inadequate ACT dose and self-medication are likely to predispose patients to unwanted malaria treatment outcomes. The Ministry of Health of Uganda instituted regulation of ACT prices in the private sector in 2016 by setting the Recommended Retail Price (RRP). However, this study found that the amount of money paid by private drug outlet clients for QAACTs was significantly different (higher) compared to the recommended retail price. This shows that the subsidies under the copayment mechanism have not been passed along to the patients in form of lower prices of QAACTs in the private sector. This is an indicator of the need to further strengthen implementation of copayment mechanism and ACT price regulation to help improve affordability and sustainability of access to QAACTs in the private sector [22].

The study had some limitations, recall bias among the drug outlet clients. This was minimized in the study by limiting the recall period to within three months. Additionally, we also reviewed the prescription notes and inspected the anti-malarial medicines that had been purchased by the study participants from the drug outlets.

## Conclusion

There is low access to QAACTs among malaria symptomatic patients in Uganda with only about a third of the study participants obtaining QAACTs in the private drug outlets. Use of sub-optimal dose of ACTs and self-medication were common among drug outlet clients who did not obtain QAACTs. Cost remains a main barrier for access to QAACTs among individuals seeking antimalarial agents in the private sector. There is need for the Ministry of Health to conduct regular monitoring the implementation of interventions to improve access to QAACTs in the country.

## Data Availability

The datasets used and/or analysed during the current study are available from the corresponding author on reasonable request.

## Abbreviations

ACT: Artemisinin-based combination therapy
AL: Artemether–lumefantrine
QAACT: Quality assured artemisinin combination therapies
AMFm: Affordable medicines facility-malaria
UNCST: Uganda National Council of Science and Technology
WHO: World Health Organization
RRP: Recommended Retail Price
WHO/HAI: World Health Organization/Health Action International
